# Evaluation of Objective Functions for the Optimal Design of an Assistive Robot

**DOI:** 10.3390/mi13122206

**Published:** 2022-12-13

**Authors:** Javier Dario Sanjuan De Caro, Md Samiul Haque Sunny, Elias Muñoz, Jaime Hernandez, Armando Torres, Brahim Brahmi, Inga Wang, Jawhar Ghommam, Mohammad H. Rahman

**Affiliations:** 1 Department of Mechanical Engineering, University of Wisconsin, Milwaukee, WI 53212, USA; 2 Department of Mechanical Engineering, Universidad del Norte, Barranquilla 081007, Colombia; 3 Computer Science, University of Wisconsin-Milwaukee, Milwaukee, WI 53212, USA; 4 Electrical Engineering Department, Collège Ahuntsic, Montreal, QC H2M 1Y8, Canada; 5 Department of Rehabilitation Sciences & Technology, University of Wisconsin-Milwaukee, Milwaukee, WI 53212, USA; 6 Electrical and Computer Engineering, Sultan Qaboos University, Muscat 123, Oman

**Keywords:** robot optimization, assistive robotics, workspace analysis, optimal design

## Abstract

The number of individuals with upper or lower extremities dysfunction (ULED) has considerably increased in the past few decades, resulting in a high economic burden for their families and society. Individuals with ULEDs require assistive robots to fulfill all their activities of daily living (ADLs). However, a theory for the optimal design of assistive robots that reduces energy consumption while increasing the workspace is unavailable. Thus, this research presents an algorithm for the optimal link length selection of an assistive robot mounted on a wheelchair to minimize the torque demands of each joint while increasing the workspace coverage. For this purpose, this research developed a workspace to satisfy a list of 18 ADLs. Then, three torque indices from the literature were considered as performance measures to minimize; the three torque measures are the quadratic average torque (QAT), the weighted root square mean (WRMS), and the absolute sum of torques (AST). The proposed algorithm evaluates any of the three torque measures within the workspace, given the robot dimensions. This proposed algorithm acts as an objective function, which is optimized using a genetic algorithm for each torque measure. The results show that all tree torque measures are suitable criteria for assistance robot optimization. However, each torque measures yield different optimal results; in the case of the QAT optimization, it produces the least workspace with the minimum overall torques of all the joints. Contrarily, the WRMS and AST optimization yield similar results generating the maximum workspace coverage but with a greater overall torque of all joints. Thus, the selection between the three methods depends on the designer’s criteria. Based on the results, the presented methodology is a reliable tool for the optimal dimensioning of assistive robots.

## 1. Introduction

The proper functioning of human upper/lower extremities is essential for personal autonomy and independence, as the upper/lower extremities are involved in every ADL. However, over the last few decades, the number of individuals with ULED has been increasing at an alarming rate. Approximately 1.7% of the US population, or 5.35 million US people, live with a central nervous system disorder that results in ULED [1]. The leading causes of ULED are spinal cord injury (SCI), amyotrophic lateral sclerosis (ALS), Cerebral Vascular Accident (CVA), trauma, amputation, and occupational injuries. From these diseases, 353,000 Americans live with an SCI (with 17,500 new cases every year), and more than 795,000 US people suffer a CVA each year (CVA with significant ULED) [1]. An estimation shows that 41 million family caregivers provide 34 billion hours of care, resulting in high costs for the families of individuals with ULED [2]. Thus, the support of most individuals living with long-term disabilities leads to severe social and economic impacts. The qualify of life of an individual relies on the proper functioning of their extremities because most of the ADLs depend either fully or partially on extremities. Consequently, the loss of extremities functions significantly limits the independence and performance of everyday tasks of the affected people. For instance, approximately 3.6 million individuals in the US use a wheelchair [2] due to ULED. The population of wheelchair users has doubled in the last decade, with an annual increase rate of 5.9% [2,3,4,5]. Furthermore, only 10% of wheelchair users report that they can complete all their ADLs [6]. Hence, those individuals need more reliable solutions to fulfill all of their ADLs.

Nowadays, robotic devices have shown immense potential to provide rehabilitation exercises, help minimize physical limitations, and increase the quality of life of people with disabilities. Nevertheless, few researchers have worked on fulfilling multiple ADLs at once; instead, they have focused on specific tasks such as mobility, transferring, and feeding. Mobility assistance robots implement a motion system for patients with ULED. An example of this type of device is the “learn empowering assistant” (LEA) [7], a mobile robot that provides support and balance for the elderly; another example is “Wheelesley” [8], a wheelchair with driving assistance. The transferring assistance robots assist patients by lifting them out of their beds or wheelchairs and moving them to new positions, e.g., a robot for interactive body assistance (RIBA) [9]. Feeding assistance robots are robots that bring assistance to individuals with ULED with eating; an example of such devices is Obi [10]. However, the mentioned robots are not capable of performing multiple ADLs. This limitation is overcome by wheelchair-mounted assistive robots, which can carry out more ADLs since they allow the user to interact with their surroundings; examples of those devices are the JACO robot [11] and the EDAN robot [12]. The main drawback of these robots is their gripper, which has issues handling small objects such as pills. Moreover, their payload capacity is low and under 1.5 kg. Thus, this reduces their coverage of essential ADLs. As a result, there is a pressing need for new robotic devices with increased load capacities and larger workspaces to cover fundamental ADLs. Thus, designers are required to design new assistive robots that overcome the limitations of the actual devices.

Researchers have employed different strategies in the design process of robots to meet application requirements. Those strategies usually rely on a proper objective function that gathers all the specific requirements for a design. Examples of such approaches include: computing the stiffness matrices of a robot to perform the topological optimization of the structure while minimizing the mass [13,14,15]. Other strategies seek to improve the manipulability of the robot by considering the product of the eigenvalues or the determinant of the Jacobian matrix, and using them to find the singular configurations of the robot [16,17,18]. In the early design stages, manipulability is the most common measure for kinematical design. Other kinematic criteria evaluate the velocity and stiffness of the robot by using the minimum eigenvalue of the Jacobian matrix [19,20,21] or computing the compliant matrix [22], respectively. However, those kinematical measures have multiple drawbacks. For example, the eigenvalues of the Jacobian matrix may be inconsistent because the kinematics of the manipulator depend on the position and orientation of the end-effector [18]. Thus, the manipulability index is suitable to compute on robots of 3-DOF with Cartesian motion [23] or in robots with positions independent of the orientation, such as PUMA [24,25]. Kim et al., in [26] proposed a methodology for standardizing the units of the Jacobian matrix to avoid issues with the eigenvalues. However, this methodology requires the assumption of an arbitrary length for the standard Jacobian matrix; since the arbitrary length can take any value, it may hide errors in the manipulator position [27,28].

Other manipulability measures use the dynamics to minimize the joint torques [29] and the energy cost [30], since minimizing joint torques produces a smoothing effect that reduces the vibrations at the end-effector [31,32] while reducing the energy cost increases the device autonomy [33,34]. Thus, diverse authors have developed different dynamic indexes for torque and energy optimization. For example, Al-Dois et al. introduced the quadratic average joint torque (QAT) to optimize a 3-DOF serial robot using a direct search method [35]. The main advantage of QAT is that it serves as a quadratic programming optimization function, which provides a global optimum if the Hessian matrix is positive definite [36]. However, the application of QAT is limited to minimizing trajectory time, not workspace optimization. Gupta et al. presented another torque measure that computes the weighted sum of the root mean square (RMS) for each joint torque through a trajectory [37]. However, the weighted sum requires additional steps such as the computation of the Pareto optimal set to determine the proper values of each weight, so that all of the values are relevant for the objective function [38]. Vidussi et al. proposed the local energy index (LEI) for task/trajectory optimization [28]. However, this measure is only applicable to trajectory optimization since it uses the motion direction of the inertia ellipsoid to compute the trajectory that minimizes the energy consumption to the desired location. Other approaches use the dynamic model of the robot and the motor parameters to obtain the total power consumption of the robot [39]. However, this measure is more suitable for trajectory optimization because it requires information about the motors and the objective position. Thus, there is no methodology that addresses the minimization of joint torques or energy consumption at the early stages for the design of assistive robots.

This work overcomes the mentioned limitations by presenting a methodology for torque minimization of assistive serial robots while increasing the workspace using three different torque measures. The three indices are adaptations of the torque measures used for the trajectory optimization, but they are modified using the proposed methodology for the workspace optimization. The torque measures are the QAT, the weighted RMS, and the absolute sum of torques (AST). The workspace used for the optimization was identified using the xArm robot and by exploring the ADLs with the highest priority. Then, a 6DOF robot is proposed and optimized for each torque measure using a genetic algorithm (GA) to find the lengths of the links that minimize the torque consumption while maximizing the workspace. Finally, we evaluated the performance of the three torque measures by comparing how much percentage of the workspace the robot can reach, and the means and standard deviations of the torque consumption of each joint within the workspace.

This paper is organized as follows: the robot and design considerations are in Section 2. Section 3 presents the methodology for the optimization using the different indices. Then, Section 4 shows the implementation of a GA to obtain the optimal dimensions of the proposed robot. Section 5 presents the results and compares the torque measures used for the optimization. Finally, the paper ends with a conclusion and future works in Section 5.

## 2. Design Considerations

### 2.1. Description of the Assistive Robot

**Figure 1:**
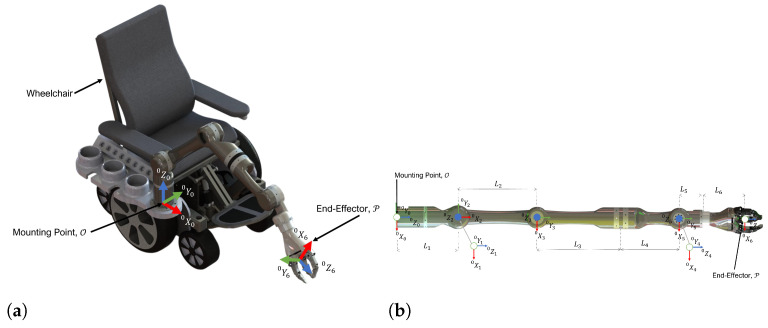
Wheelchair-mounted assistive robot. (**a**) CAD, (**b**) Joint Coordinate Definition.

In this research, a wheelchair-mounted 6DOF robot is considered to provide ADL assistance to individuals with ULED. The proposed robot is a serial manipulator that consists of six revolute joints. The Denavit Hartenberg (DH) parameters corresponding to the robot configuration shown in Figure 1b are presented in Table 1. The homogeneous transformation matrix (HTM) [40] derived from the DH parameters is expressed as follows:

(1) $${\;}^{i-1}T_{i}=\left[\begin{array}{cccc} 1 & 0 & 0 & a_{i-i}\\ 0 & c\alpha_{i-i} & -s\alpha_{i-i} & 0\\ 0 & s\alpha_{i-i} & c\alpha_{i-i} & 0\\ 0 & 0 & 0 & 1 \end{array}\right]\left[\begin{array}{cccc} c\theta_{i} & -s\theta_{i} & 0 & 0\\ s\theta_{i} & c\theta_{i} & 0 & 0\\ 0 & 0 & 1 & d_{i}\\ 0 & 0 & 0 & 1 \end{array}\right]$$

$$\begin{array}{cc} \; & \alpha_{i-1}=\mathrm{link}\;\mathrm{twist}\\ \; & a_{i-1}=\mathrm{link}\;\mathrm{length}\\ \; & \theta_{i}=\mathrm{joint}\;\mathrm{variable}\;\mathrm{of}\;\mathrm{revolute}\;\mathrm{joint}\\ \; & d_{i}=\mathrm{link}\;\mathrm{offset}\;\mathrm{and}/\mathrm{or}\;\mathrm{joint}\;\mathrm{variable}\;\mathrm{of}\;\mathrm{prismatic}\;\mathrm{joint} \end{array}$$

As shown in Figure 1a, the base frame O of the robot is located at the point *O*, at the mounting plate of the robot.

### 2.2. Inverse Kinematics

The inverse kinematics is the computation of the joint-angles, given the pose of the end-effector. This process requires the solution of a non-linear set of trigonometric equations. Many methods are available in the state-of-the-art to find the analytical solution of the inverse kinematic problem, including algebraic methods [41], geometrical approaches [42,43,44], and Buchberger’s algorithm [45], among others. The inverse kinematic solution requires a lot of steps that go beyond the scope of this research. Thus, we present the result of the inverse kinematic solution of the proposed 6DOF robot, as follows:

(2) $$\begin{array}{cc} {\;}^{0}P_{5} & ={\;}^{0}P_{6}-\left(L_{5}+L_{6}\right){\;}^{0}R_{6}\left[\begin{array}{c} 0\\ 0\\ 1 \end{array}\right]\\ \theta_{1} & =atan\left(\frac{{\;}^{0}P_{5,y}}{{\;}^{0}P_{5,x}}\right)\\ \theta_{2} & =atan\left(\frac{\sqrt{{\;}^{0}P_{5,x}^{2}+{\;}^{0}P_{5,y}^{2}}}{{\;}^{0}P_{5,z}-L_{1}}\right)\\ & -asin\left(\frac{\left(L_{3}+L_{4}\right)c\theta_{3}}{\sqrt{{\;}^{0}P_{5,x}^{2}+{\;}^{0}P_{5,y}^{2}+{\left({\;}^{0}P_{5,z}-L_{1}\right)}^{2}}}\right)\\ \theta_{3} & =acos\left(\frac{-L_{2}^{2}-{\left(L_{3}+L_{4}\right)}^{2}+{\;}^{0}P_{5,x}^{2}+{\;}^{0}P_{5,y}^{2}+{\left({\;}^{0}P_{5,z}-L_{1}\right)}^{2}}{2L_{2}\left(L_{3}+L_{4}\right)}\right)\\ \theta_{4} & =atan\left(\frac{\kappa_{1}}{yc\theta_{1}-xs\theta_{1}}\right)\\ \theta_{5} & =atan\left(\frac{\sqrt{\kappa_{2}}}{2\kappa_{3}}\right)\\ \theta_{6} & =atan\left(\frac{\kappa_{4}}{\kappa_{5}}\right) \end{array}$$

The terms $\kappa_{1}$ to $\kappa_{5}$ are presented in Appendix A.1.

### 2.3. Jacobian Matrix

The variation in the pose of the end-effector is related to the variation in the joints angles using the Jacobian matrix. Thus, the Jacobian matrix is presented as follows:

(3) $$J=\left[\begin{array}{cccc} \frac{\partial P_{x}}{\partial \theta_{1}} & \frac{\partial P_{x}}{\partial \theta_{2}} & \cdots & \frac{\partial P_{x}}{\partial \theta_{6}}\\ \frac{\partial P_{y}}{\partial \theta_{1}} & \frac{\partial P_{y}}{\partial \theta_{2}} & \cdots & \frac{\partial P_{y}}{\partial \theta_{6}}\\ \frac{\partial P_{z}}{\partial \theta_{1}} & \frac{\partial P_{z}}{\partial \theta_{2}} & \cdots & \frac{\partial P_{z}}{\partial \theta_{6}}\\ \frac{\partial \theta_{x}}{\partial \theta_{1}} & \frac{\partial \theta_{x}}{\partial \theta_{2}} & \cdots & \frac{\partial \theta_{x}}{\partial \theta_{6}}\\ \frac{\partial \theta_{y}}{\partial \theta_{1}} & \frac{\partial \theta_{y}}{\partial \theta_{2}} & \cdots & \frac{\partial \theta_{y}}{\partial \theta_{6}}\\ \frac{\partial \theta_{z}}{\partial \theta_{1}} & \frac{\partial \theta_{z}}{\partial \theta_{2}} & \cdots & \frac{\partial \theta_{z}}{\partial \theta_{6}} \end{array}\right]$$

where *P*, $\theta_{x},\theta_{y},$ and $\theta_{z}$ are the position and orientation obtained from the successive multiplication of the HTM obtained from the DH parameters. The $\theta_{i}$ represents each joint angle.

### 2.4. Assistive Robot Model

**Figure 2:**
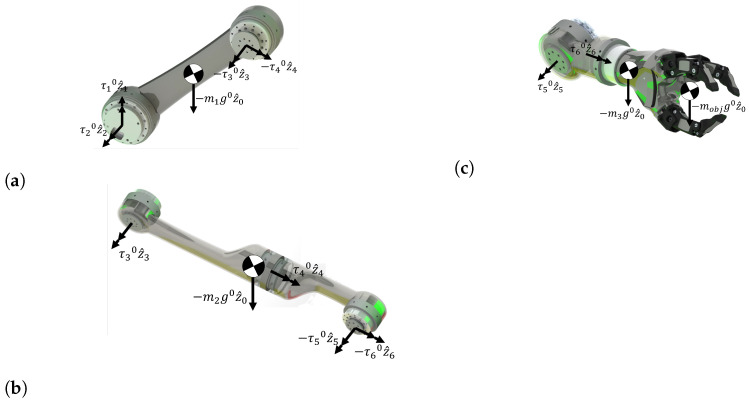
Free body diagrams of the simplified links. (**a**) Link 1, (**b**) Link 2, (**c**) Link 3.

The dynamic model of the proposed assistive robot is obtained using the virtual work method and the principle of D’alembert for the free body diagrams presented in Figure 2. However, we found that a complete dynamical was impractical because the torque due to the inertial effects was negligible for this application. Section 5 tests this assumption, showing the results of a simulation of the trajectory of the assistive robot. Thus, the torque of each is computed using a static model as follows:

(4) $$\begin{array}{cc} \tau & =\left[\begin{array}{c} 0\\ \kappa_{6}\\ \kappa_{7}\\ \kappa_{8}\\ \kappa_{9}\\ 0 \end{array}\right]\\ \; & =\hat{G} \end{array}$$

The $\kappa_{6}$ to $\kappa_{9}$ terms are presented in Appendix A.2. Equation (4) represents the effects of gravity on each joint due to the mass of each link. Furthermore, the effects of a payload ($M_{obj}$) or a moving object at the end-effector of the robot are included by computing the Jacobian matrix at the end-effector (*J*) and adding the virtual work performed by the payload. The following equation presents the static model, including the effects of a payload at the end-effector:

(5) $$\tau =\hat{G}+J^{T}W_{ef}$$

where $W_{ef}$ is the wrench produced by the moving object at the end-effector. The following equation presents $W_{ef}$:

(6) $$W_{ef}=\left[\begin{array}{c} 0\\ 0\\ M_{obj}g\\ 0\\ 0\\ 0 \end{array}\right]$$

Equation (5) is the static model of the assistive robot.

### 2.5. ADLs Workspace

This section seeks to define the workspace that will include the majority of ADLs or the ADLS with the highest priority. Thus, we consider the reasons for ULED users to utilize assistive robots. According to [5], individuals with ULED are motivated to use assistive robots that provide aids to accomplish essential ADLs, such as eating, drinking, and picking/placing objects. Moreover, Huete et al. in [46] performed a study with the ASIBOT that incorporated task prioritization. This study found that people with a disability, especially with ULED, prioritize personal hygiene activities, such as combing hair, applying makeup, and brushing teeth. In addition, they consider activities such as manipulating objects, eating, and drinking as being secondary. Furthermore, Bedaf et al. in [47] reported that their participants preferred aids in activities such as mobility, self-care, and social isolation. Thus, based on the different approaches to identify essential ADLs, we proposed the following list of ADLs:

- Maneuvering spoon/fork to take food from bowl to plate,
- Holding/maneuvering spoon/fork to put food in the mouth,
- Holding cup near the mouth, and gradual upward positioning of the cup during drinking,
- Holding medicine,
- Opening/closing the refrigerator or oven door,
- Push–pull, swing open–close, automatic door opener button for doors,
- Picking/placing objects from/on a table,
- Opening and closing drawers,
- Picking/placing objects from the upper shelf,
- Picking up an object from the ground,
- Opening/closing the lid of a jar, box, paper box, or cap of bottles,
- Holding printed books or turning pages,
- Removing a hanger from the closet,
- Holding credit cards, swiping credit cards at the market, or ATM booth,
- Holding a pen, maneuvering on paper or surfaces,
- Holding the phone near the ear, or putting in a speakerphone,
- Brushing teeth,
- Applying makeup.

**Figure 3:**
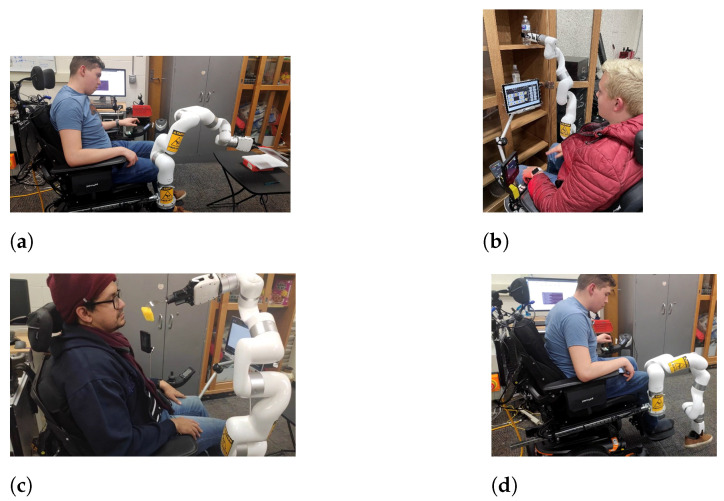
Activities of daily living achieved with different control systems. (**a**) Picking/placing an object from a table. (**b**) Picking an object from upper shelf. (**c**) Eating with a fork. (**d**) Picking up an object from the ground.

**Figure 4:**
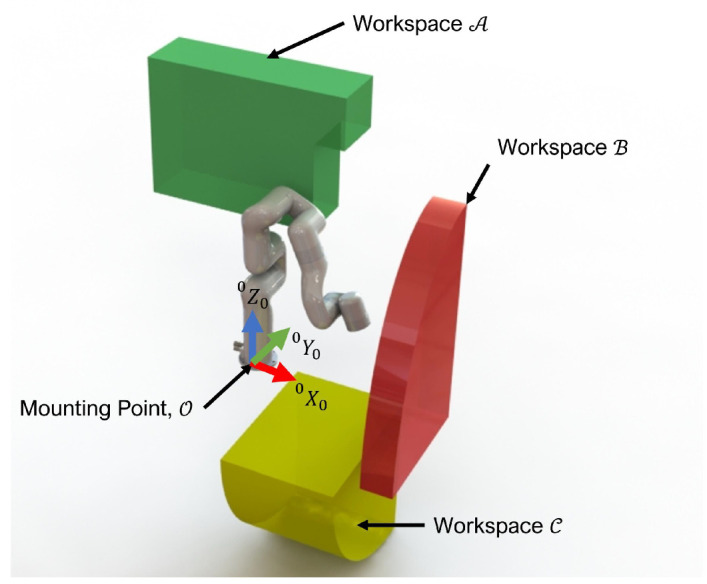
Required Workspace for the essential ADLs.

According to the approved study protocols, participants aged over 18 gave their written consent to conduct experiments with a commercially available 6DoF robot (xArm6) [48]. Figure 3 shows examples where the users are maneuvering the xArm6 robot and performing ADL tasks. Thanks to the results from the experiments, we obtained the workspace needed to conduct the above-listed ADLs tasks establishing three different workspaces (Workspace-A, Workspace-B, and Workspace-C) as presented in Figure 4. The sum of all the sections represents the desired workspace of the robot, i.e., the workspace that covers all of the essential ADLs. Although the desired workspace presented is not continuous, in reality, a robot that can reach the three mentioned workspaces can make the transition between them. Table 2 details the three workspaces and the corresponding ADLs tasks for each region. Thus, workspace A (a green workspace in Figure 4) is the space used for activities related to holding and maneuvering things near the user. Workspace B (the red workspace) is far from the wheelchair and covers the workspace from a surface of a desk table until the upper shelf of a kitchen cabinet. Workspace C (a yellow workspace) covers the space next to the wheelchair, and is used to pick objects from the ground. Additionally, we established a required orientation based on the ADLs for each workspace. Thus, workspace A requires the alignment of the end-effector with the positive *y*-axis for activities such as holding a spoon. Workspace B requires the end-effector to be aligned with the positive x-axis to pick objects from a table or a shelf. Finally, the end-effector at workspace C points to the negative *z*-axis for holding items from the floor.

## 3. Algorithm for Torque Computation within the Workspace

**Figure 5:**
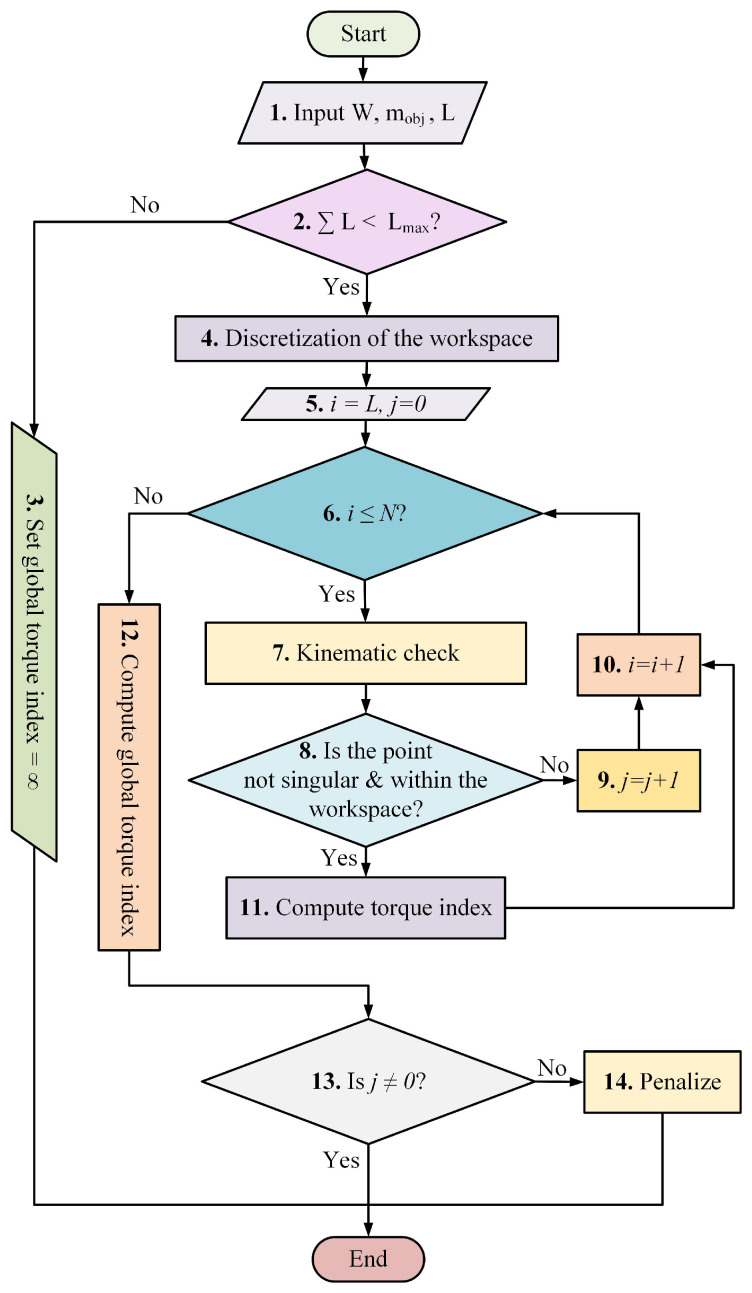
Flowchart of the algorithm used to compute the objective function within the workspace.

The proposed algorithm for torque minimization is presented in Figure 5. Given a torque measure, the link lengths of the robot, and a workspace, this algorithm computes the torque within the given workspace. The workspace was discretized to facilitate the evaluation of the torque measure. Moreover, the algorithm evaluates each position within the workspace using inverse kinematics and the Jacobian to determine singularities. Each step of the algorithm is presented in the following subsections.

### 3.1. Inputs

This section corresponds to steps 1 to 3 of the algorithm. In those steps, the algorithm asks the user for three variables, *W*, $m_{obj}$, and $\vec{L}$, where *W* represents the whole workspace, which is the sum of the three workspaces described in Section 2.5. Thus, *W* is expressed as follows:

(7) $$\{W_{A},W_{B},W_{C}\}\in W$$

$m_{obj}$ represents the weight of the carried object. $\vec{L}$ corresponds to a vector with all the link lengths as presented below:

(8) $$\vec{L}=\left[\begin{array}{c} L_{1}\\ L_{2}\\ L_{3}\\ L_{4}\\ L_{5}\\ L_{6} \end{array}\right]$$

These link lengths are the same as those used in the DH parameters (see Table 1). Additionally, we added a link-length constraint expressed by Equation (9) to avoid infinitely long lengths.

(9) $$\sum_{i=1}^{6}L_{i}<L_{max}$$

Thus, if the sum of the robot link lengths is longer than $L_{max}$, the algorithm assigns a value of infinity to the global torque index of step 12. Otherwise, the algorithm goes to step 4. Note that by infinity, we mean a really big value; in the case of using a 64 bits computer, the values assigned is $2^{64}$.

### 3.2. Discretization of the Workspace

The discretization of the workspace is step 4 of the algorithm. Considering the workspaces presented in Section 2.5, the bounds of each workspace are obtained, given that they are rectangular prims and segmented cylinders. Then, the bounds of the axis of each workspace are expressed as $\left[X_{min},X_{max}\right]$, $\left[Y_{min},Y_{max}\right]$, and $\left[Z_{min},Z_{max}\right]$ for the rectangular prims; and $\left[X_{min},X_{max}\right]$, $\left[R_{min},R_{max}\right]$, and $\left[\theta_{min},\theta_{max}\right]$ for the segmented cylinders. Then, the following equation is used to segment each interval:

(10) $$p_{k}=p_{min}+\frac{\left(p_{max}-p_{min}\right)\left(k-1\right)}{n_{w}}$$

where $n_{w}$ is the number of segments for an axis of a workspace. $p_{min}$ and $p_{max}$ are the lower and upper bounds of an axis. $p_{k}$ is the $k^{th}$ segment of an axis, with $k=1,2,\cdots ,n_{w}$. The boundaries for each workspace are shown in Table 3. Once the intervals are segmented, the Cartesian product of the interval $\left[X_{min},X_{max}\right]\times \left[Y_{min},Y_{max}\right]\times \left[Z_{min},Z_{max}\right]$ (workspace A) and $\left[X_{min},X_{max}\right]\times \left[R_{min},R_{max}\right]\times \left[\theta_{min},\theta_{max}\right]$ (workspaces B and C) are computed, obtaining $n_{w}^{3}$ points distributed within each workspace. Finally, when considering the complete desired workspace (*W*), the total number of points is obtained as follows:

(11) $$N=\sum_{w=1}^{3}n_{w}^{3}$$

### 3.3. Workspace Sweeping

The workspace sweeping corresponds to steps 5 to 9 of the algorithm. In step 5, the algorithm initializes two counters, *i* and *j*; the former represents a point *i* within *W* ($p_{i}$). The counter *i* increases within the *for loop* started at step 5, until the complete workspace is analyzed (i≤N). Note that each configuration of vector $\vec{L}$ generates a different workspace; thus, the counter *j* counts the points within *W* that are not within the reachable workspace of the robot or are singular. The algorithm detects if a point is out of the reachable workspace of the robot via the utilization of the inverse kinematics (Equation (2)). In addition, the algorithm detects singularities within the workspace if the determinant of the Jacobian is zero. This strategy also satisfies the manipulability requirements of the robot [49,50]. Then, if any of these two conditions are satisfied, the algorithm goes to step 9, increasing the counter *j* by one. On the other hand, if none of the two conditions are satisfied, the algorithm goes to step 11. Section 3.4 explains step 11. Finally, at step 10, the algorithm increases the counter *i* by one, then returns to step 6, repeating the cycle.

### 3.4. Local Torque Measures

Step 11 is defined for three different torque indices, the QAT, the weighted RMs, and the AST. Since the implementation of the algorithm depends on which index is being evaluated, the three indices are presented separately as follows:

#### 3.4.1. Quadratic Average Torque (QAT)

The QAT is defined as the quadratic average of torques using the following equation:

$$QAT=\int_{0}^{T}\sum_{joint=1}^{n_{j}}{\left(\frac{\tau_{joint}\left(t\right)}{\tau_{joint}^{\max}}\right)}^{2}dt$$

where *T* is the trajectory time. In this context, $\tau_{joint}$ represents the torque of the ${joint}^{th}$ of the robot, and $n_{j}$ represents the total number of joints. However, as explained in [35], this measure is used for trajectory generation. Thus, for link lengths optimization, the QAT is computed locally as a position *i* of the workspace, using the following expression:

(12) $$QAT_{i}=\sum_{joint=1}^{n_{j}}\left(\frac{\tau_{joint}}{\tau_{joint}^{\max}}\right)$$

#### 3.4.2. Weighted Root Mean Square (WRMS)

The weighted sum as presented in [37] requires the calculation of each individual joint torque within the workspace. Then, the following equation is applied to compute the WRMS:

(13) $$WRMS=\sum_{joint=1}^{n_{j}}w_{joint}\tau_{joint,rms}$$

where $w_{joint}$’s is a weight assigned to each joint torque. The weights are selected such that their sum is equal to 1, and, according to [37], each torque uses the same weight for each joint torque $\tau_{joint}$. Note that Equation (13) requires to know the torque of the ${joint}^{th}$ of the robot through the trajectory. Then, to apply the WRMS, at step 11 the values of each individual torque are collected in an array for position *i* of the workspace, as follows:

(14) $$\tau_{joint}=\left[\begin{array}{c} \tau_{joint-1}\\ \tau_{joint-2}\\ \vdots \\ \tau_{joint-i}\\ \vdots \\ \tau_{joint-N} \end{array}\right]$$

#### 3.4.3. The Absolute Sum of Torques (AST)

In [39], the authors presented a index for measuring the energy consumption in a trajectory. The index is expressed as follows:

(15) $$E_{robot}=\int_{o}^{t}W_{r}\left(t\right)dt$$

where $W_{r}$ is the robot energy consumption, obtained by the sum of the individual energy consumption of each joint, as follows:

(16) $$W_{r}\left(t\right)=\sum_{joint=1}^{6}W_{m,joint}\left(t\right)$$

The individual energy consumption of each joint ($W_{m,i}$) is obtained by using the following equation:

(17) $$W_{m,joint}\left(t\right)=\omega_{joint}\tau_{joint}$$

Assuming that the angular velocity of each joint ($\omega_{i}$) is unitary, Equation (15) is simplified to obtain the following expression for the local AST at position *i* of the workspace:

(18) $$AST_{i}=\sum_{joint=1}^{n_{j}}\left|\tau_{i}\right|$$

Lastly, note that the indices presented in Equations (12), (14) and (18) are computed at the position *i* of the workspace if step 8 is satisfied. Thus, in the case that the robot is at a singular position or is not in the reachable workspace, the value of the local index is zero.

### 3.5. Global Torque Index Computation

The global torque index computation refers to step 12 of the algorithm. The computation of the global index for the QAT and AST requires similar equations, which are presented as follows:

(19) $$QAT_{global}=\sum_{i=1}^{N}QAT_{i}$$

(20) $$AST_{global}=\sum_{i=1}^{N}AST_{i}$$

Lastly, in the case of WRMS, the global index is computed using Equation (13), where the RMS of each joint is computed as follows:

(21) $$\tau_{joint,rms}=\sqrt{\frac{\tau_{joint-1}^{2}+\tau_{joint-2}^{2}+\cdots +\tau_{joint-N}^{2}}{N}}$$

### 3.6. Penalization

This section corresponds to steps 13 and 14 of the algorithm. The penalization scheme punishes the configurations of $\vec{L}$ that fail to cover completely *W*, i.e., j≠0. Thus, if j≠0, the algorithm implements the following penalization equation to the GSTI:

(22) $$\bar{Global}={\left(\frac{N}{N-j}\right)}^{2}Global$$

where Global represents any of the indexes presented in Equations (19), (20) or (13). $\bar{Global}$ is any of the global indexes after penalization. This way, if Global is small but the counter *j* is high, the penalization increases the value of $\bar{GSTI}$. This way, the optimization will avoid the configurations that yield a minuscule workspace.

## 4. Optimization of a 6DOF Assistive Robot

**Figure 6:**
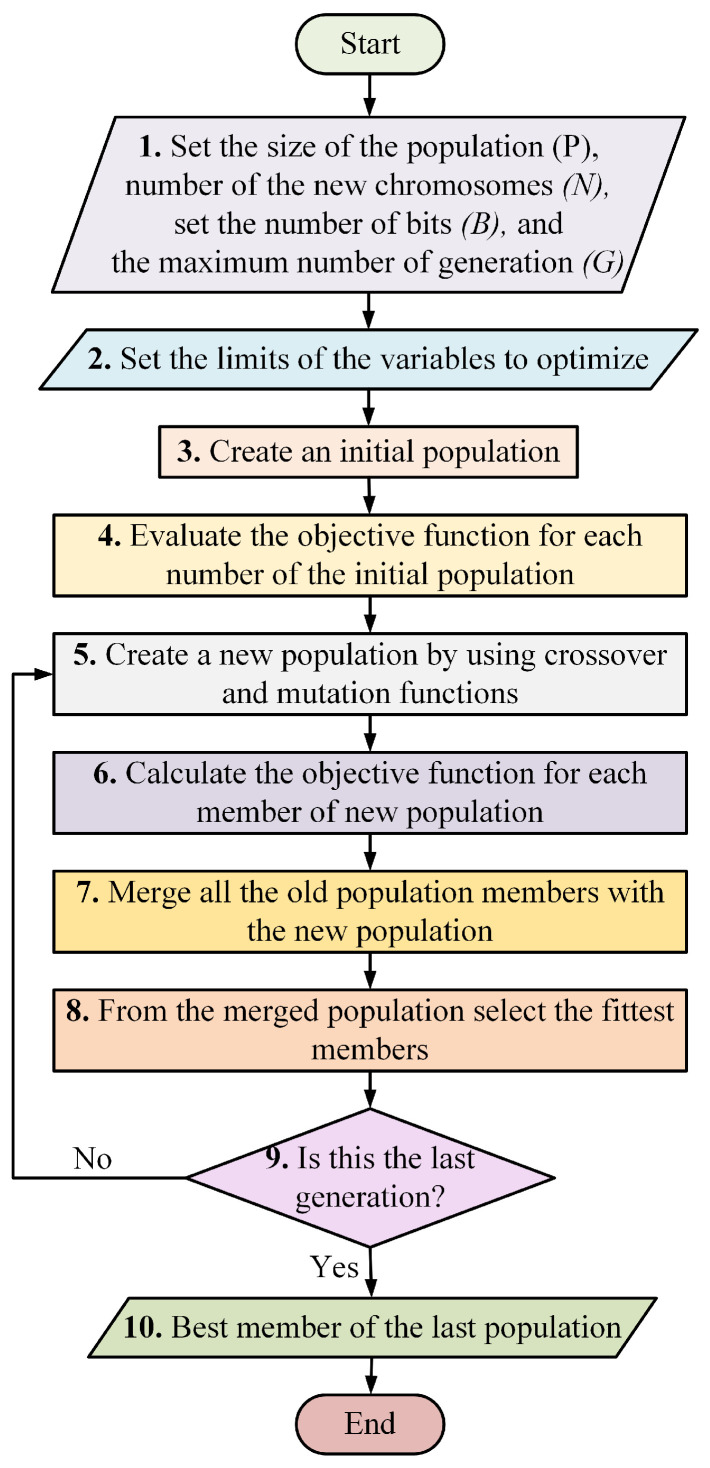
Flowchart of the genetic algorithm.

The objective of the optimization is the selection of the link lengths that minimize the torque consumption of the robot while maintaining the desired workspace. For this purpose, the algorithm presented in Section 3 is used as the objective function for each torque measure. The optimization is carried away using GA. The reasons for selecting GA is its outstanding global optimization ability and parallel computing capabilities. In addition, GA is a heuristic method that uses statistics and evolutionary theory to find the most suitable solution for an objective function [51]. Figure 6 presents a flowchart to explain the GA for optimization.

Table 4 presents the robot parameters and the constants required for the utilization of the algorithm. The constants P, B, N, and G were selected to assure the proper convergence of a GA based on the experimental results of [52,53]. Each robot link has an interval for each length. The lower bound represents the minimum distance required to assemble each link of the robot and the space needed to attach each motor. The upper bound assures the correct implementation of the GA, allowing the GA to explore a broad number of values. $L_{max}$ was selected based on the sizes of robots with similar capabilities, such as the JACO arm. The mass of the carrying object ($m_{obj}$) is double the load capacity of the commercial assistive robots available [11]. Finally, the linear density $\rho_{l}$ considers that the links are made of carbon fiber and have a diameter of 50mm, with a thickness of 4mm. The implementation of the linear density influences the values of the output torque from the static model, as a high length will increase the mass of each link.

## 5. Results and Discussion

**Figure 7:**
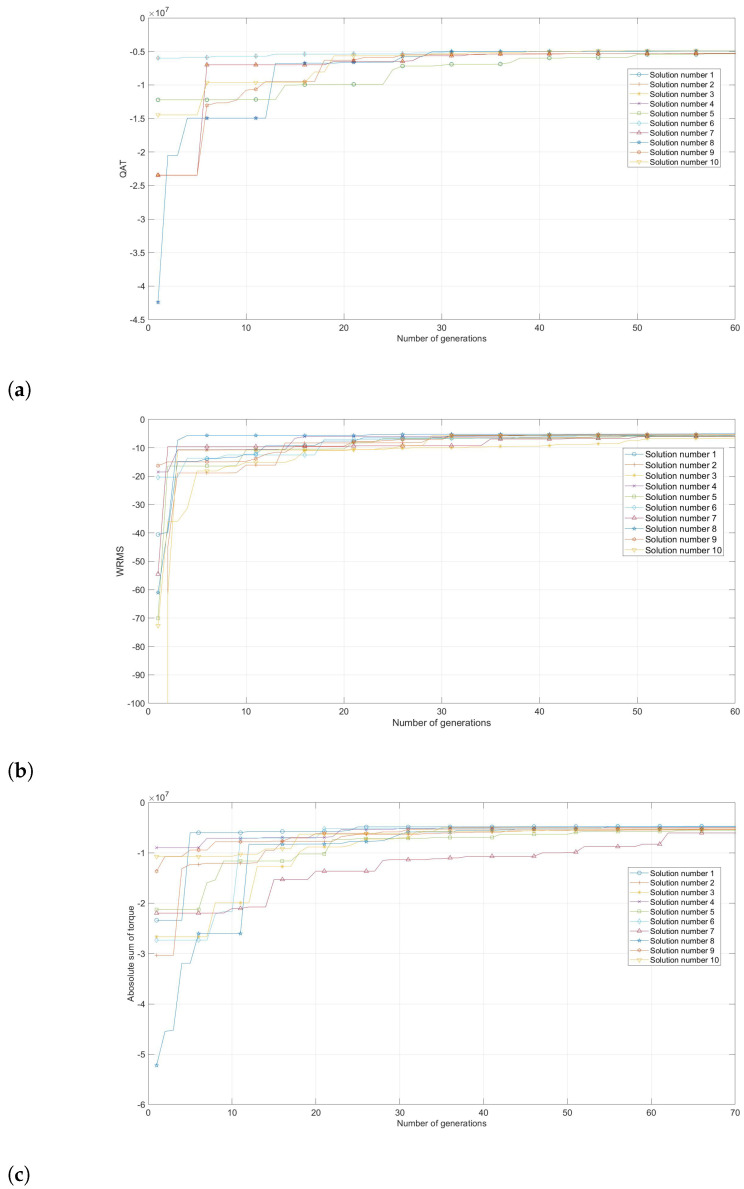
Evolution of the each index from 10 random initial generations. (**a**) QAT, (**b**) WMRS, (**c**) AST.

Table 5 presents the results from the GA optimization for the three torque measures. The results are the vector $\vec{L}$ for each torque measure and the evaluation of each torque measure with each $\vec{L}$. Note that the value of QAT is minimum for the $\vec{L}$ obtained from the QAT optimization compared to the QAT yield via the optimization using WRMS or AST. A similar result is acquired when considering the $\vec{L}$ obtained from WRMS and AST optimization. Thus, this result implies that the three indices reached different and independent optimal results. Figure 7 shows the evolution of each index from 10 random initial link lengths. The figure clearly shows that each index converged to a single solution, which suggests that each index reached the global optimum. However, since the three indices achieved three different optimal solutions, the question of which index yields the highest workspace while reducing the torque consumption is still unanswered.

To answer this question, consider Table 6, which presents the mean (μ), standard deviation (σ), and maximum value of torque for each joint of the robot through the workspace for each optimal result, including the workspace coverage. It is clear that AST generates the highest workspace coverage, but with the maximum mean and maximum torque for each joint. Opposite to this result, the QAT optimization yields the least workspace coverage but with the minimum torque consumption. The results of the WRMS are comparable to the results of the AST because both indices generate similar results. Thus, from the presented results, we conclude that all of the indices are suitable for the optimal link length design of the assistive robot, but the selection of which index will depend on which are the designer’s priorities. In this way, if the designer selects QAT, a smaller workspace but with less torque consumption will be obtained. Contrary, if the designer uses AST or WRMS, this will generate a broader workspace with a higher torque consumption.

**Figure 8:**
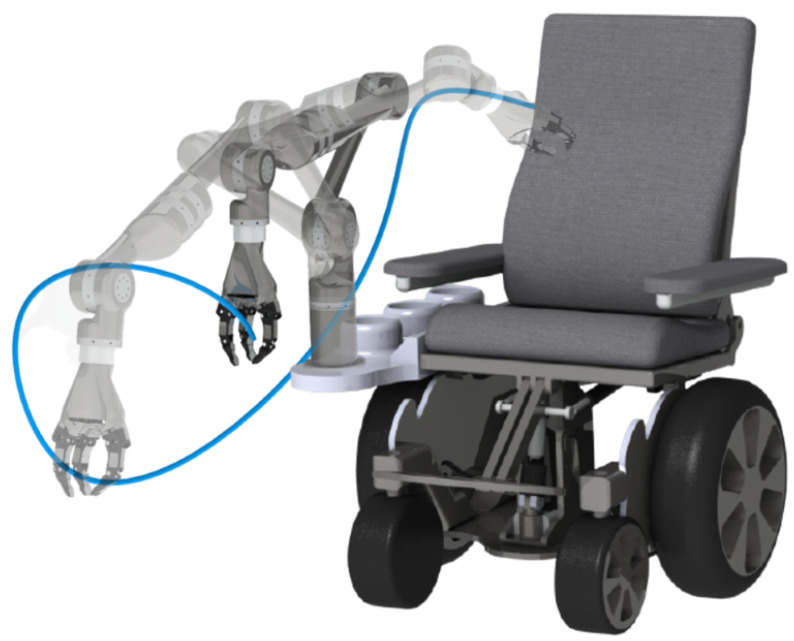
Trajectory for testing the assumption of static torque instead of dynamic torque.

**Figure 9:**
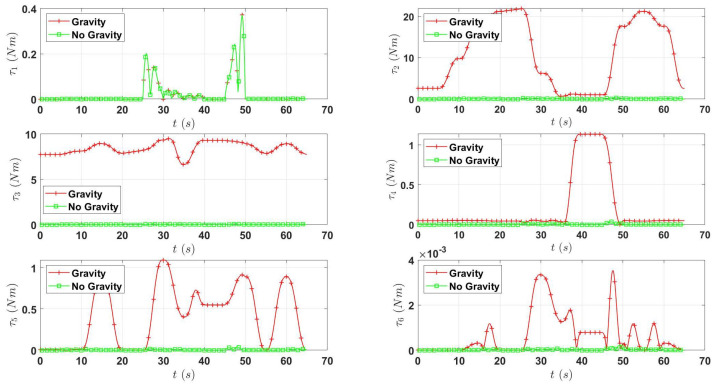
Plot of the torque generated for a trajectory for each joint.

Lastly, the assumption of using only the gravity effects to compute the torque is tested by plotting the torque of each joint for the trajectory presented in Figure 8. Then, the plot considers the torques generated in the trajectory with and without the gravity. These results are presented in Figure 9; as can be seen, the inertial effects are almost negligible compared to the contribution of the torque generated due to gravity. Thus, this validates the assumption of Equation (4).

## 6. Conclusions

This paper presents a comparative analysis of three different torque measures for the optimal design of an assistive robot mounted on a wheelchair. The three measures are the QAT, WRMS, and AST, and they measure the torque distribution within the robot workspace. The three indices are modifications of the torque indices used for selecting robot trajectories, as found in the literature. Moreover, the article presents a methodology for the link length optimization of robots. The proposed approach requires the definition of a desirable workspace for the assistive robot, which is obtained for different ADLs via experimentation with the xArm robot. The workspace is a tool to evaluate the workspace coverage and the computation of the indices for different link length configurations of the assistive robot. A penalization function is added to the methodology to increase the performance indices when the obtained workspace is too short relative to the desired workspace. Then, the three indices are optimized for the proposed assistive robot using GA, generating three optimal values. The optimal solutions were compared, obtaining that the solutions using the AST and the WRMS are equivalent and yield the widest workspace but the highest torque consumption. In contrast, the optimal solution using the QAT is the opposite, producing a smaller workspace but with less torque consumption. Thus, the three torque measures produce valid optimal solutions. Finally, we conclude that the presented algorithm is a reliable tool for designing serial robots since the elements considered in this study are not limited to assistive robots. Future works will try to expand the ideas of this paper to include optimization of the DH parameters.

## Figures and Tables

**Table 1:** DH Parameters of the Assistive Robot.

	$a_{i-1}$	$\alpha_{i-1}$	$d_{i}$	$\theta_{i}$
Joint 1	0	0	$L_{1}$	$\theta_{1}$
Joint 2	0	$\frac{\pi }{2}$	0	$\theta_{2}+\frac{\pi }{2}$
Joint 3	$L_{2}$	0	0	$\theta_{3}-\frac{\pi }{2}$
Joint 4	0	$-\frac{\pi }{2}$	$L_{3}+L_{4}$	$\theta_{4}$
Joint 5	0	$\frac{\pi }{2}$	0	$\theta_{5}$
Joint 6	0	$-\frac{\pi }{2}$	$L_{5}+L_{6}$	$\theta_{6}$

**Table 2:** Aimed ADLs of each workspace.

	ADLs Tasks
Workspace A	Holding/maneuvering spoon/fork to put food in the mouth; Holding cup near the mouth and gradual upward positioning of the cup during drinking; Holding the phone near the ear, or putting in speakerphone; Brushing teeth; Applying makeup
Workspace B	Push–pull, swing open–close, automatic door opener button doors; Holding Pen, maneuvering on paper or surfaces; Maneuvering spoon/fork to take food from bowl to plate; Picking/placing objects from the upper shelf
Workspace C	Picking up an object from the ground

**Table 3:** Boundaries of the Workspaces with respect to the origin O. Dimensions in mm and angles in deg.

	$X_{min}$	$X_{max}$	$Y_{min}$	$Y_{max}$	$Z_{min}$	$Z_{max}$
$W_{A}$	−460	0	0	470	330	800
	$X_{min}$	$X_{max}$	$R_{min}$	$R_{max}$	$\theta_{min}$	$\theta_{max}$
$W_{B}$	550	650	0	430	90	0
$W_{C}$	140	500	0	500	−180	−90

**Table 4:** Optimization parameters.

Required Constants	Values
P	200
B	10
N	200
G	150
$L_{1}$ limits	$\left[200.0,\;500.0\right]\;\mathrm{mm}$
$L_{2}$ limits	$\left[150.0,\;500.0\right]\;\mathrm{mm}$
$L_{3}$ limits	$\left[150.0,\;500.0\right]\;\mathrm{mm}$
$L_{4}$ limits	$\left[100.0,\;500.0\right]\;\mathrm{mm}$
$L_{5}$ limits	$\left[130.0,\;500.0\right]\;\mathrm{mm}$
$L_{6}$ limits	$\left[150.0,\;500.0\right]\;\mathrm{mm}$
$L_{max}$	1260.4mm
$m_{obj}$	3kg
$\rho_{l}$	1.0116kg/m
$n_{k}$	40

**Table 5:** Comparison of link lengths and optimal values.

Torque Measure		Optimum Lengths	Torque Measure Values		
	**(mm)**		**QAT**	**WRMS**	**AST**
QAT	$L_{1}$	211.437	$4.69\times 10^{6}$	5.16	$5.91\times 10^{6}$
	$L_{2}$	458.9443			
	$L_{3}$	154.4477			
	$L_{4}$	114.0762			
	$L_{5}$	133.9785			
	$L_{6}$	152.0528			
WRMS	$L_{1}$	217.5953	$4.72\times 10^{6}$	4.9865	$5.89\times 10^{6}$
	$L_{2}$	412.4145			
	$L_{3}$	233.1378			
	$L_{4}$	117.2043			
	$L_{5}$	130			
	$L_{6}$	150			
AST	$L_{1}$	239.8827	$4.70\times 10^{6}$	5.0207	$5.87\times 10^{6}$
	$L_{2}$	412.4145			
	$L_{3}$	192.4242			
	$L_{4}$	135.5816			
	$L_{5}$	130			
	$L_{6}$	150			

**Table 6:** Comparison of the mean, standard deviation, and maximum torque, and the workspace coverage of each optimal result.

Index	Joints	$\tau_{\mu }$ (Nm)	$\tau_{\sigma }$ (NM)	Maximum	Workspace Coverage
QAT	joint 1	0	0	0	93.10%
	joint 2	6.7918771	4.809291138	16.4754774	
	joint 3	4.531285416	3.059260467	14.7946312	
	joint 4	3.889101148	2.808458665	8.01195021	
	joint 5	3.865609749	2.7771379	8.01195127	
	joint 6	0	0	0	
WRMS	joint 1	0	0	0	96.02%
	joint 2	7.118705693	4.805923665	16.5240628	
	joint 3	5.910992298	3.326024889	16.5792462	
	joint 4	3.665542598	2.807587818	7.85134041	
	joint 5	3.883523338	2.802911663	7.85138995	
	joint 6	0	0	0	
AST	joint 1	0	0	0	96.20%
	joint 2	7.150709607	4.817382775	16.5092107	
	joint 3	6.357707317	3.441201661	17.3769848	
	joint 4	3.474306559	2.739109293	7.85134994	
	joint 5	4.115075621	2.8968119	7.85138995	
	joint 6	0	0	0	

## Data Availability

The datasets generated during and/or analyzed during the current study are not publicly available due to the conditions of the funding source, but are available from the corresponding author upon reasonable request.

## Appendix A

### Appendix A.1. Coefficients Equation (2)

The following equations expresses the coefficients $k_{1}$ to $k_{6}$ of Equation (2).

$$\begin{array}{cc} \kappa_{1} & =L_{1}\;c\left(\theta_{2}\right)\;s\left(\theta_{3}\right)+L_{1}\;c\left(\theta_{3}\right)\;s\left(\theta_{2}\right)\\ \; & -z\;c\left(\theta_{2}\right)\;s\left(\theta_{3}\right)-z\;c\left(\theta_{3}\right)\;s\left(\theta_{2}\right)\\ \; & +L_{2}\;{c\left(\theta_{2}\right)}^{2}\;s\left(\theta_{3}\right)\\ & +L_{2}\;{c\left(\theta_{1}\right)}^{2}\;{s\left(\theta_{2}\right)}^{2}\;s\left(\theta_{3}\right)\\ \; & +L_{2}\;{s\left(\theta_{1}\right)}^{2}\;{s\left(\theta_{2}\right)}^{2}\;s\left(\theta_{3}\right)\\ & +L_{2}\;c\left(\theta_{2}\right)\;c\left(\theta_{3}\right)\;s\left(\theta_{2}\right)\\ \; & -x\;c\left(\theta_{1}\right)\;c\left(\theta_{2}\right)\;c\left(\theta_{3}\right)\\ & -y\;c\left(\theta_{2}\right)\;c\left(\theta_{3}\right)\;s\left(\theta_{1}\right)\\ \; & +x\;c\left(\theta_{1}\right)\;s\left(\theta_{2}\right)\;s\left(\theta_{3}\right)\\ & +y\;s\left(\theta_{1}\right)\;s\left(\theta_{2}\right)\;s\left(\theta_{3}\right)\\ \; & +L_{3}\;c\left(\theta_{2}\right)\;{c\left(\theta_{3}\right)}^{2}\;s\left(\theta_{2}\right)\\ & +L_{3}\;{c\left(\theta_{2}\right)}^{2}\;c\left(\theta_{3}\right)\;s\left(\theta_{3}\right)\\ \; & -L_{3}\;c\left(\theta_{2}\right)\;s\left(\theta_{2}\right)\;{s\left(\theta_{3}\right)}^{2}\\ & -L_{3}\;c\left(\theta_{3}\right)\;{s\left(\theta_{2}\right)}^{2}\;s\left(\theta_{3}\right)\\ \; & -L_{2}\;{c\left(\theta_{1}\right)}^{2}\;c\left(\theta_{2}\right)\;c\left(\theta_{3}\right)\;s\left(\theta_{2}\right)\\ & -L_{2}\;c\left(\theta_{2}\right)\;c\left(\theta_{3}\right)\;{s\left(\theta_{1}\right)}^{2}\;s\left(\theta_{2}\right)\\ \; & -L_{3}\;{c\left(\theta_{1}\right)}^{2}\;c\left(\theta_{2}\right)\;{c\left(\theta_{3}\right)}^{2}\;s\left(\theta_{2}\right)\\ & -L_{3}\;{c\left(\theta_{1}\right)}^{2}\;{c\left(\theta_{2}\right)}^{2}\;c\left(\theta_{3}\right)\;s\left(\theta_{3}\right)\\ \; & -L_{3}\;c\left(\theta_{2}\right)\;{c\left(\theta_{3}\right)}^{2}\;{s\left(\theta_{1}\right)}^{2}\;s\left(\theta_{2}\right)\\ & +L_{3}\;{c\left(\theta_{1}\right)}^{2}\;c\left(\theta_{2}\right)\;s\left(\theta_{2}\right)\;{s\left(\theta_{3}\right)}^{2}\\ \; & +L_{3}\;{c\left(\theta_{1}\right)}^{2}\;c\left(\theta_{3}\right)\;{s\left(\theta_{2}\right)}^{2}\;s\left(\theta_{3}\right)\\ & -L_{3}\;{c\left(\theta_{2}\right)}^{2}\;c\left(\theta_{3}\right)\;{s\left(\theta_{1}\right)}^{2}\;s\left(\theta_{3}\right)\\ \; & +L_{3}\;c\left(\theta_{2}\right)\;{s\left(\theta_{1}\right)}^{2}\;s\left(\theta_{2}\right)\;{s\left(\theta_{3}\right)}^{2}\\ & ]]3\;c\left(\theta_{3}\right)\;{s\left(\theta_{1}\right)}^{2}\;{s\left(\theta_{2}\right)}^{2}\;s\left(\theta_{3}\right) \end{array}$$

$$\begin{array}{cc} \kappa_{2} & =y^{2}\;c\left(2\;\theta_{1}\right)-2\;{L_{2}}^{2}\;c\left(2\;\theta_{3}\right)-x^{2}\;c\left(2\;\theta_{1}\right)\\ & -4\;L_{1}\;z+\frac{x^{2}\;c\left(2\;\theta_{2}-2\;\theta_{1}+2\;\theta_{3}\right)}{2}\\ & +\frac{x^{2}\;c\left(2\;\theta_{1}+2\;\theta_{2}+2\;\theta_{3}\right)}{2}\\ \; & -2\;{L_{1}}^{2}\;c\left(2\;\theta_{2}+2\;\theta_{3}\right)\\ & -\frac{y^{2}\;c\left(2\;\theta_{2}-2\;\theta_{1}+2\;\theta_{3}\right)}{2}\\ \; & -\frac{y^{2}\;c\left(2\;\theta_{1}+2\;\theta_{2}+2\;\theta_{3}\right)}{2}+x^{2}\;c\left(2\;\theta_{2}+2\;\theta_{3}\right)\\ \; & +y^{2}\;c\left(2\;\theta_{2}+2\;\theta_{3}\right)-2\;z^{2}\;c\left(2\;\theta_{2}+2\;\theta_{3}\right)\\ & +2\;{L_{1}}^{2}+2\;{L_{2}}^{2}+3\;x^{2}+3\;y^{2}\\ \; & +2\;z^{2}-4\;L_{1}\;L_{2}\;c\left(\theta_{2}+2\;\theta_{3}\right)\\ & +2\;L_{1}\;y\;c\left(\theta_{1}+2\;\theta_{2}+2\;\theta_{3}\right)\\ \; & -2\;L_{2}\;y\;c\left(\theta_{2}-\theta_{1}+2\;\theta_{3}\right)\\ & -2\;L_{1}\;x\;s\left(\theta_{1}+2\;\theta_{2}+2\;\theta_{3}\right)\\ & -2\;L_{2}\;x\;s\left(\theta_{2}-\theta_{1}+2\;\theta_{3}\right)\\ \; & +2\;L_{2}\;y\;c\left(\theta_{1}-\theta_{2}\right)+4\;L_{2}\;z\;c\left(\theta_{2}+2\;\theta_{3}\right)\\ & -2\;y\;z\;c\left(\theta_{1}+2\;\theta_{2}+2\;\theta_{3}\right)\\ \; & -2\;L_{2}\;x\;s\left(\theta_{1}-\theta_{2}\right)+2\;x\;z\;s\left(\theta_{1}+2\;\theta_{2}+2\;\theta_{3}\right)\\ & -2\;L_{1}\;y\;c\left(2\;\theta_{2}-\theta_{1}+2\;\theta_{3}\right)\\ \; & -2\;L_{1}\;x\;s\left(2\;\theta_{2}-\theta_{1}+2\;\theta_{3}\right)\\ & +4\;L_{1}\;z\;c\left(2\;\theta_{2}+2\;\theta_{3}\right)\\ \; & +2\;y\;z\;c\left(2\;\theta_{2}-\theta_{1}+2\;\theta_{3}\right)-2\;x\;y\;s\left(2\;\theta_{1}\right)\\ \; & -x\;y\;s\left(2\;\theta_{2}-2\;\theta_{1}+2\;\theta_{3}\right)\\ & +x\;y\;s\left(2\;\theta_{1}+2\;\theta_{2}+2\;\theta_{3}\right)\\ & +2\;x\;z\;s\left(2\;\theta_{2}-\theta_{1}+2\;\theta_{3}\right)\\ \; & +2\;L_{2}\;y\;c\left(\theta_{1}+\theta_{2}+2\;\theta_{3}\right)\\ & -2\;L_{2}\;x\;s\left(\theta_{1}+\theta_{2}+2\;\theta_{3}\right)\\ \; & +4\;L_{1}\;L_{2}\;c\left(\theta_{2}\right)-2\;L_{2}\;y\;c\left(\theta_{1}+\theta_{2}\right)\\ & +2\;L_{2}\;x\;s\left(\theta_{1}+\theta_{2}\right)-4\;L_{2}\;z\;c\left(\theta_{2}\right) \end{array}$$

$$\begin{array}{cc} \kappa_{3} & =z\;c\left(\theta_{2}+\theta_{3}\right)-L_{4}\\ & -\frac{x\;s\left(\theta_{1}+\theta_{2}+\theta_{3}\right)}{2}-L_{1}\;c\left(\theta_{2}+\theta_{3}\right)\\ \; & -\frac{y\;c\left(\theta_{2}-\theta_{1}+\theta_{3}\right)}{2}\\ & -\frac{x\;s\left(\theta_{2}-\theta_{1}+\theta_{3}\right)}{2}-L_{2}\;c\left(\theta_{3}\right)\\ \; & -L_{3}+\frac{y\;c\left(\theta_{1}+\theta_{2}+\theta_{3}\right)}{2} \end{array}$$

$$\begin{array}{cc} \kappa_{4} & =c\left(\theta_{4}\right)\;s\left(\theta_{1}\right)+\sqrt{\kappa_{6}}+\\ & c\left(\theta_{1}\right)\;c\left(\theta_{2}\right)\;c\left(\theta_{3}\right)\;s\left(\theta_{4}\right)\\ \; & -c\left(\theta_{1}\right)\;s\left(\theta_{2}\right)\;s\left(\theta_{3}\right)\;s\left(\theta_{4}\right) \end{array}$$

$$\begin{array}{cc} \kappa_{5} & =c\left(\theta_{5}\right)\;s\left(\theta_{1}\right)\;s\left(\theta_{4}\right)\\ & -\left(c\theta_{y}c\theta_{z}\right)+c\left(\theta_{1}\right)\;c\left(\theta_{2}\right)\;s\left(\theta_{3}\right)\;s\left(\theta_{5}\right)\\ \; & +c\left(\theta_{1}\right)\;c\left(\theta_{3}\right)\;s\left(\theta_{2}\right)\;s\left(\theta_{5}\right)\\ & -c\left(\theta_{1}\right)\;c\left(\theta_{2}\right)\;c\left(\theta_{3}\right)\;c\left(\theta_{4}\right)\;c\left(\theta_{5}\right)\\ \; & +c\left(\theta_{1}\right)\;c\left(\theta_{4}\right)\;c\left(\theta_{5}\right)\;s\left(\theta_{2}\right)\;s\left(\theta_{3}\right) \end{array}$$

$$\begin{array}{cc} \kappa_{6} & =-{\left(c\theta_{y}c\theta_{z}\right)}^{2}\\ & +{c\left(\theta_{1}\right)}^{2}\;{c\left(\theta_{2}\right)}^{2}\;{c\left(\theta_{3}\right)}^{2}\;{c\left(\theta_{4}\right)}^{2}\;{c\left(\theta_{5}\right)}^{2}\\ \; & +{c\left(\theta_{1}\right)}^{2}\;{c\left(\theta_{2}\right)}^{2}\;{c\left(\theta_{3}\right)}^{2}\;{s\left(\theta_{4}\right)}^{2}\\ \; & -2\;{c\left(\theta_{1}\right)}^{2}\;{c\left(\theta_{2}\right)}^{2}\;c\left(\theta_{3}\right)\;c\left(\theta_{4}\right)\;c\left(\theta_{5}\right)\;s\left(\theta_{3}\right)\;s\left(\theta_{5}\right)\\ \; & +{c\left(\theta_{1}\right)}^{2}\;{c\left(\theta_{2}\right)}^{2}\;{s\left(\theta_{3}\right)}^{2}\;{s\left(\theta_{5}\right)}^{2}\\ \; & -2\;{c\left(\theta_{1}\right)}^{2}\;c\left(\theta_{2}\right)\;{c\left(\theta_{3}\right)}^{2}\;c\left(\theta_{4}\right)\;c\left(\theta_{5}\right)\;s\left(\theta_{2}\right)\;s\left(\theta_{5}\right)\\ \; & -2\;{c\left(\theta_{1}\right)}^{2}\;c\left(\theta_{2}\right)\;c\left(\theta_{3}\right)\;{c\left(\theta_{4}\right)}^{2}\;{c\left(\theta_{5}\right)}^{2}\;s\left(\theta_{2}\right)\;s\left(\theta_{3}\right)\\ \; & -2\;{c\left(\theta_{1}\right)}^{2}\;c\left(\theta_{2}\right)\;c\left(\theta_{3}\right)\;s\left(\theta_{2}\right)\;s\left(\theta_{3}\right)\;{s\left(\theta_{4}\right)}^{2}\\ \; & +2\;{c\left(\theta_{1}\right)}^{2}\;c\left(\theta_{2}\right)\;c\left(\theta_{3}\right)\;s\left(\theta_{2}\right)\;s\left(\theta_{3}\right)\;{s\left(\theta_{5}\right)}^{2}\\ \; & +2\;{c\left(\theta_{1}\right)}^{2}\;c\left(\theta_{2}\right)\;c\left(\theta_{4}\right)\;c\left(\theta_{5}\right)\;s\left(\theta_{2}\right)\;{s\left(\theta_{3}\right)}^{2}\;s\left(\theta_{5}\right)\\ \; & +{c\left(\theta_{1}\right)}^{2}\;{c\left(\theta_{3}\right)}^{2}\;{s\left(\theta_{2}\right)}^{2}\;{s\left(\theta_{5}\right)}^{2}\\ \; & +2\;{c\left(\theta_{1}\right)}^{2}\;c\left(\theta_{3}\right)\;c\left(\theta_{4}\right)\;c\left(\theta_{5}\right)\;{s\left(\theta_{2}\right)}^{2}\;s\left(\theta_{3}\right)\;s\left(\theta_{5}\right)\\ \; & +{c\left(\theta_{1}\right)}^{2}\;{c\left(\theta_{4}\right)}^{2}\;{c\left(\theta_{5}\right)}^{2}\;{s\left(\theta_{2}\right)}^{2}\;{s\left(\theta_{3}\right)}^{2}\\ \; & +{c\left(\theta_{1}\right)}^{2}\;{s\left(\theta_{2}\right)}^{2}\;{s\left(\theta_{3}\right)}^{2}\;{s\left(\theta_{4}\right)}^{2}\\ \; & -2\;c\left(\theta_{1}\right)\;c\left(\theta_{2}\right)\;c\left(\theta_{3}\right)\;c\left(\theta_{4}\right)\;{c\left(\theta_{5}\right)}^{2}\;s\left(\theta_{1}\right)\;s\left(\theta_{4}\right)\\ \; & +2\;c\left(\theta_{1}\right)\;c\left(\theta_{2}\right)\;c\left(\theta_{3}\right)\;c\left(\theta_{4}\right)\;s\left(\theta_{1}\right)\;s\left(\theta_{4}\right)\\ \; & +2\;c\left(\theta_{1}\right)\;c\left(\theta_{2}\right)\;c\left(\theta_{5}\right)\;s\left(\theta_{1}\right)\;s\left(\theta_{3}\right)\;s\left(\theta_{4}\right)\;s\left(\theta_{5}\right)\\ \; & +2\;c\left(\theta_{1}\right)\;c\left(\theta_{3}\right)\;c\left(\theta_{5}\right)\;s\left(\theta_{1}\right)\;s\left(\theta_{2}\right)\;s\left(\theta_{4}\right)\;s\left(\theta_{5}\right)\\ \; & +2\;c\left(\theta_{1}\right)\;c\left(\theta_{4}\right)\;{c\left(\theta_{5}\right)}^{2}\;s\left(\theta_{1}\right)\;s\left(\theta_{2}\right)\;s\left(\theta_{3}\right)\;s\left(\theta_{4}\right)\\ \; & -2\;c\left(\theta_{1}\right)\;c\left(\theta_{4}\right)\;s\left(\theta_{1}\right)\;s\left(\theta_{2}\right)\;s\left(\theta_{3}\right)\;s\left(\theta_{4}\right)\\ \; & +{c\left(\theta_{4}\right)}^{2}\;{s\left(\theta_{1}\right)}^{2}+{c\left(\theta_{5}\right)}^{2}\;{s\left(\theta_{1}\right)}^{2}\;{s\left(\theta_{4}\right)}^{2} \end{array}$$

### Appendix A.2. Coefficients Equation (4)

The following equations expresses the coefficients $k_{7}$ to $k_{10}$.

$$\begin{array}{cc} \kappa_{7} & =-g\;\frac{2\;L_{2}\;m_{1}\;s\left(\theta_{2}\right)+4\;L_{2}\;m_{2}\;s\left(\theta_{2}\right)+4\;L_{2}\;m_{3}\;s\left(\theta_{2}\right)+2\;L_{3}\;m_{2}\;s\left(\theta_{2}+\theta_{3}\right)}{4}\\ \; & -g\frac{4\;L_{3}\;m_{3}\;s\left(\theta_{2}+\theta_{3}\right)+2\;L_{4}\;m_{2}\;s\left(\theta_{2}+\theta_{3}\right)+4\;L_{4}\;m_{3}\;s\left(\theta_{2}+\theta_{3}\right)}{4}\\ \; & -g\frac{L_{5}\;m_{3}\;c\left(\theta_{2}+\theta_{3}\right)\;s\left(\theta_{4}+\theta_{5}\right)+L_{6}\;m_{3}\;c\left(\theta_{2}+\theta_{3}\right)\;s\left(\theta_{4}+\theta_{5}\right)}{4}\\ \; & -g\frac{2\;L_{5}\;m_{3}\;s\left(\theta_{2}+\theta_{3}\right)\;c\left(\theta_{5}\right)+2\;L_{6}\;m_{3}\;s\left(\theta_{2}+\theta_{3}\right)\;c\left(\theta_{5}\right)}{4}\\ \; & +g\frac{L_{5}\;m_{3}\;s\left(\theta_{4}-\theta_{5}\right)\;c\left(\theta_{2}+\theta_{3}\right)-L_{6}\;m_{3}\;s\left(\theta_{4}-\theta_{5}\right)\;c\left(\theta_{2}+\theta_{3}\right)}{4} \end{array}$$

$$\begin{array}{cc} \kappa_{8} & =-\frac{L_{3}\;g\;m_{2}\;s\left(\theta_{2}+\theta_{3}\right)}{2}-L_{3}\;g\;m_{3}\;s\left(\theta_{2}+\theta_{3}\right)\\ \; & -\frac{L_{4}\;g\;m_{2}\;s\left(\theta_{2}+\theta_{3}\right)}{2}-L_{4}\;g\;m_{3}\;s\left(\theta_{2}+\theta_{3}\right)\\ \; & -\frac{L_{5}\;g\;m_{3}\;s\left(\theta_{2}+\theta_{3}\right)\;c\left(\theta_{5}\right)}{2}-\frac{L_{6}\;g\;m_{3}\;s\left(\theta_{2}+\theta_{3}\right)\;c\left(\theta_{5}\right)}{2}\\ \; & -\frac{L_{5}\;g\;m_{3}\;c\left(\theta_{2}+\theta_{3}\right)\;c\left(\theta_{4}\right)\;s\left(\theta_{5}\right)}{2}-\frac{L_{6}\;g\;m_{3}\;c\left(\theta_{2}+\theta_{3}\right)\;c\left(\theta_{4}\right)\;s\left(\theta_{5}\right)}{2} \end{array}$$

$$\kappa_{9}=\frac{g\;m_{3}\;s\left(\theta_{2}+\theta_{3}\right)\;s\left(\theta_{4}\right)\;s\left(\theta_{5}\right)\;\left(L_{5}+L_{6}\right)}{2}$$

$$\begin{array}{cc} \kappa_{10} & =-g\;m_{3}\;\left(L_{5}+L_{6}\right)\;\frac{c\left(\theta_{2}\right)\;c\left(\theta_{3}\right)\;s\left(\theta_{5}\right)-s\left(\theta_{2}\right)\;s\left(\theta_{3}\right)\;s\left(\theta_{5}\right)}{2}\\ \; & -g\;m_{3}\;\frac{c\left(\theta_{2}\right)\;c\left(\theta_{4}\right)\;c\left(\theta_{5}\right)\;s\left(\theta_{3}\right)+c\left(\theta_{3}\right)\;c\left(\theta_{4}\right)\;c\left(\theta_{5}\right)\;s\left(\theta_{2}\right)}{2} \end{array}$$
